# Stammering

**Published:** 1908-12-05

**Authors:** P. Macleod Yearsley

**Affiliations:** Senior Surgeon the Royal Ear Hospital


### Hospital Clinics.

### STAMMERING.

By P. MACLEOD YEARSLEY, F.R.C.S., Senior Surgeon the Royal Ear Hospital.

(Report of a Lecture delivered at the Polyclinic.)

The phenomena and treatment of stammering are not as a rule much considered by medical men, although their advice is often asked about the matter. In straying out of the beaten track in the subject of this lecture, it is with the idea of enabling you to deal fully with the condition, if so be you have a patient thus affected.

In order properly to understand the production of stammering it is necessary first of all to know the mechanisms by which speech is produced. Speech depends upon the exact co-ordination of two mechanisms, the laryngeal and the oral. Below these two is a third, the respiratory mechanism, about which I will say a few words at once. First, the respiratory pressure of air is provided continuously during speech for each clause or sentence. Secondly, it is made to vary for the required effects of emphasis and expression. Thirdly, there is a cortical centre, which innervates the respiratory efforts, but which has not yet been definitely localised.

The vocal and oral mechanisms are each capable of being employed separately, though for speaking purposes they must be accurately co-ordinated. The classical illustration of this necessity for co-ordination is the comparison of speaking with playing on the violin. Skill in playing this instrument depends on the accurate co-ordination of the two hands, the bow hand and the string hand, which can be compared with the vocal and oral mechanisms of speech. Among learners it is not uncommon to find that the bow hand fails to produce sound because it has not been properly employed owing to excessive attention to the string hand. So in speech, stammering may be caused by failure of the oral centre to modify that which is produced by the vocal mechanism.

You may compare the voiced and voiceless elements of speech by considering such words as *satisfy*, *wonder*, *wonderful*. In the case of the first the larynx has to produce three separate vowel sounds, and between them it has to open the vocal cords so as to allow air to pass into the mouth where the consonants s, t, and f are pronounced. By contrast, with the word *wonder* every letter contains voice, and therefore the vocal cords must be together throughout the word; but in the adjective *wonderful* they must separate in order to allow the formation of the voiceless f of the last syllable.

It is advantageous in any discussion of the causes of stammering to consider the physiological alphabet, and to compare it with the ordinary alphabet of the philologist. Every letter sound is a product of the laryngeal and oral efforts combined, but the vowels are more purely laryngeal in origin than the consonants. In the passage through the mouth the overtones of the vowel sounds are suppressed or exaggerated, and this is what causes the differentiation of the different vowels. The most convenient order in which to consider the vowel sounds is to take them as they are formed by the modified mouth, in order from the narrowest to the broadest. This order, if the letters are given the Latin pronunciation, is i, e, a, o, u. Now if to these vowels you add friction from the mouth you convert them more or less into consonantal forms. We used to be told at school that w and y are sometimes vowels; but they are not pure vowels, though they are (sometimes) vowels to which mouth friction has been added, as in words beginning with w in which that letter has the effect of a prolonged oo.

Of the consonants there are three main varieties, voiced, voiceless, and naso-resonant, and they are largely produced at three main situations which are called stop positions. The first great stop position is at the lips, and is called the anterior or labial Here are produced the voiceless explosive p, and the voiced explosive b, which is merely p with a little voice added to it from the larynx. At this stop position are also formed the nasal resonant m, which is pronounced in the same way as b, except that the soft palate is dropped and the air allowed to escape through the nose. This explains how it is that in nasal obstruction m is pronounced like b. The voiced fricative formed here is w, as in wood; and the voiceless fricative w, as in what.

The second stop position for the formation of consonants is known as the anterior linguo-palatal. Just as with the labial position, there are five consonants formed here. The complete stop is effected by applying the tongue into close contact with the hard palate just behind the teeth. The first consonant formed there is the explosive t; this with voice added gives d. The nasal resonant formed here is n; the voiced fricative is sh (as in *she*), and the voiceless fricative is zh, as in *pleasure*. This last sound is not used in English at the beginning of a word, but is common in French, for instance, *Jean*, *Georges*. The letter r is produced by a kind of purring friction set up in the air by the tip of the tongue; and the letter l is formed in the same way, but over the sides of the tongue instead of the tip.

The third stop position is the posterior linguo-palatal, where once more five consonants are pronounced, by coaptation of the tongue to the back of the palate. The voiceless explosive in this position is k; this sound is effected by contact of the tongue and palate fairly far forwards in such a word as *key*, further back in *coal*. The corresponding voiced explosive is g, and the nasal resonant is ng, at the end of a word as in *wing*. The voiced fricative is here y in *yes*, and the voiceless fricative is h. There is a good deal of difference of opinion as to how exactly h is produced, and it has even been suggested that to drop one's h's is really a form of stammering.

There are two other stop positions which must be briefly mentioned. The labiodental position is where f and v are produced. The other is the linguodental where th, voiced and voiceless, are formed as in *thy*

and *thin* respectively. I must just for a moment, before I take up the consideration of stammering itself, compare this alphabet with the ordinary one taught in schools. Omitting the letters which do not exist in the physiological alphabet, the order is a b d e f h i k l m n o p r s t u v w y z. Thus c is omitted because it is always either s or k. Again, q has no value without a following u, and qu is exactly identical with kw. Similarly x is merely ks.

The starting point of stammering is a *want of promptitude* of the vocal element of speech. Generally speaking, there is least difficulty with vowels, and this explains why stammerers can always sing without stammering. So, also, when starting with a consonant, the difficulty is not so much to "touch off" the consonant properly and accurately as to bring up the next vocal sound after it. In ignorance of where the fault lies, the stammerer devotes more and more effort to his consonant, and less and less to the following letter.

In order to test and differentiate the letters and to find out where the difficulty of pronunciation arises, a very useful list of test sentences has been drawn up by Wyllie. As a test for vowels consider this sentence: "Eels ail amid ocean ooze." Next we have a sentence which tests the voiced fricatives: "We visit the Zulus like ramblers yearly." Voiceless consonants are tested by another sentence: "Far shores seem thinly hazy." For nasal resonants a sufficient test is: "My nephew." For the voiced explosives "Best gold dust" is used; and for the voiceless explosives: "Two poor comrades." These sentences represent the order of difficulty for stammerers in most cases, but not in all.

It is, as I have said, want of promptitude in the vocal mechanism which is primarily the basis of stammering. This want of promptitude may be a lagging of the voice during the production of the first syllable; or the voice may be not merely lagging, but feeble in quality as well. Owing to inefficient respiratory mechanism you may find cases in which there is a marked sense of fatigue after speaking. Or the laryngeal mechanism may be so badly managed that the speaker breaks from the normal baritone or bass into a falsetto. Lastly, there is the fault of endeavouring to speak not during expiration, but during inspiration. This is called drawback phonation.

In the oral mechanism the faults are due mostly to a surcharge of energy: the stutterer may stick at his fricatives, or there may be an overflow of energy into spasmodic and involuntary movements of other parts of the body; sometimes facial contortions, sometimes movements of the limbs, as when it is only by stamping with the foot that the word can be uttered. In a few cases the energy flows into the upper non-vocal part of the larynx. If during the phonation the false cords are thus closed over the true, the sufferer struggles with cyanosed face endeavouring to free his larynx and get air. This has been called by Kussmal the gutturo-tetanic form of stammering.

It is remarkable that males should suffer so much more often than do females; the proportion is something like four to one. As a rule the defect begins in childhood spontaneously, but sometimes it is picked up by imitation of another sufferer, and becomes a habit. It is especially common in those of neuropathic inheritance, though it must be understood that there is nothing whatever of mental deficiency associated with it. Fright or violent emotion have been known to set up stammering, which is nearly always worse in the presence of strangers.

The prognosis depends largely upon the stammerer's own intelligent co-operation. The best age at which to begin treatment is twelve to sixteen, as by then the patient is capable of understanding the explanation of his defects. When stammering is complicated by severe spasmodic movements the prognosis is rather less good: and if the intellect be in any way defective it is distinctly bad.

Treatment must be systematic, and based upon the particular factors at work. It is important to impress on the patient that the vocal mechanism of the larynx is at fault. He should be made to read aloud for not less than ten minutes a day: this reading should be poetry to commence with, because it requires more voice than does prose.

To cultivate the singing voice is also useful; but it is not wise to teach the patient to sing everything, otherwise he gets into the habit of speaking always in a sing-song way. Test the speech with the sentences already given, and thus find out on what letters the greatest difficulty is experienced. If you find that a patient stammers on initial vowels, his exercises should be confined to these for weeks on end. Consonants in such a case must be attempted only after the vowels have been mastered.

If there is any tendency to attempt speech with an empty chest, instruction must be given to avoid the habit, and here respiratory exercises are particularly useful. In cases of especial difficulty it is generally wisest to place the patient with a tutor or a specialist. Last of all, with the exception of the correction of nasal obstruction, there is no surgical operation which has the slightest effect upon stammering; and the practice of declamation with the mouth full of pebbles, recommended by the example of Demosthenes, is one to be very strongly deprecated.

